# Tackling Control of a Cosmopolitan Phytopathogen: *Sclerotinia*

**DOI:** 10.3389/fpls.2021.707509

**Published:** 2021-08-20

**Authors:** Cathryn A. O’Sullivan, Katharina Belt, Louise F. Thatcher

**Affiliations:** ^1^CSIRO Agriculture and Food, St Lucia, QLD, Australia; ^2^CSIRO Agriculture and Food, Floreat, WA, Australia; ^3^CSIRO Agriculture and Food, Acton, ACT, Australia

**Keywords:** sclerotia, necrotroph, fungal pathogen, biocontrol, biopesticide, fungicide, horticulture, row crops

## Abstract

Phytopathogenic members of the *Sclerotinia* genus cause widespread disease across a broad range of economically important crops. In particular, *Sclerotinia sclerotiorum* is considered one of the most destructive and cosmopolitan of plant pathogens. Here, were review the epidemiology of the pathogen, its economic impact on agricultural production, and measures employed toward control of disease. We review the broad approaches required to tackle *Sclerotinia* diseases and include cultural practices, crop genetic resistance, chemical fungicides, and biological controls. We highlight the benefits and drawbacks of each approach along with recent advances within these controls and future strategies.

## Introduction

*Sclerotinia* rot, also referred to as white mould on some crops, is a widespread fungal disease caused by phytopathogenic members of the *Sclerotinia* genus. In particular, *S. sclerotiorum* is considered one of the most destructive and cosmopolitan of plant pathogens (Bolton et al., 2006; Saharan and Mehta, 2008; Smolinska and Kowalska, 2018). *Sclerotinia* is widely distributed throughout temperate regions but also occurs in more arid areas. A lack of adequate host genetic resistance, the wide host range of the pathogen, and the general difficulty, both culturally and chemically, in managing the disease are the main drivers for *Sclerotinia* species causing extensive crop damage within both broad acre and horticultural farming sectors. Economic losses result from collapsed vegetable crops that are completely unmarketable, and in grain or oilseed crops as a reduction in seed number, weight, or quality (Bolton et al., 2006; Saharan and Mehta, 2008; Peltier et al., 2012; Derbyshire and Denton-Giles, 2016). Sclerotia, the resting stage of the fungus, can also contaminate harvested seed, reducing seed price because of the detection of foreign material in the product (Peltier et al., 2012).

*Sclerotinia* rots can be caused by three closely related species: *S. sclerotiorum*, *Sclerotinia Trifoliorum*, and *Sclerotinia minor*. Combined, they are known to infect over 500 plant species, mostly from Dicotyledonae but a few are from Monocotyledonae such as onion and garlic (Laemmlen, 2001; Saharan and Mehta, 2008; Willbur et al., 2019). *S. sclerotiorum* has a wide host range, and *S. minor* affects similar hosts but within a reduced range. The host range of *S. trifoliorum* is narrower, usually, only forage legumes such as clover and lucerne/alfalfa. Numerous broadleaf weeds such as wild clover, dandelion, capeweed, and wild radish are also hosts and play a role in carry-over of the disease and inoculum between crops. Although *Sclerotinia* diseases are typically a sporadic production problem, the disease has become a consistent issue in intensive cropping systems with short rotations. The drivers for increased disease emergence include favourable environmental/seasonal conditions such as reliable rainfall or irrigation, and the frequency of susceptible hosts in the cropping rotation, which build up levels of soil-borne sclerotia.

Economically important broad acre crops commonly affected by *S. sclerotiorum* include the oilseed crops canola and sunflower, and the legumes soybean, peanuts, chickpea, and several bean species, and its infection often leads to a significant loss in crop production (Agrios, 2005; Link and Johnson, 2007; Saharan and Mehta, 2008; Derbyshire and Denton-Giles, 2016). The incidence of *Sclerotinia* stem rot on canola is problematic worldwide and reported in most canola-producing regions of the world such as China, India, Europe, Australia, and North and South America (reviewed in Alkooranee et al., 2017; Zheng et al., 2020). In the United States, collective crop losses to *S. sclerotiorum* exceed US$200 million annually (Bolton et al., 2006). In 2010, the disease cost Canada an estimated US$600M in lost canola production (Dupont Pioneer Report, 2012). In smaller production areas such as Australia, losses estimated over AUS$59M have been recorded (Khangura et al., 2014). In canola, diseases can be widespread but highly sporadic, requiring specific environmental conditions to develop. As such, disease incidence can vary greatly from year to year, but is most damaging under prolonged wet conditions, particularly leading up to and during flowering and where the crop is grown intensively, resulting in a build-up of pathogen load, in the form of sclerotia, within the cropping system (Khangura et al., 2014, 2015; Derbyshire and Denton-Giles, 2016). Under conducive conditions, yield losses typically exceed 20–35%, but incidences of over 50 and up to 80–100% have been reported in some global markets (Markell et al., 2009; Dokken-Bouchard et al., 2010; Murray and Brennan, 2012; reviewed in Alkooranee et al., 2017). In Canada, it is predicted that for every 1% of canola crop infections, there is a 0.5% loss in potential yield results (Derbyshire and Denton-Giles, 2016).

In legumes, years that favour the development of *Sclerotinia* diseases are a significant cause of economic losses. The disease is considered “chronic to epidemic” on soybean throughout the world with the worst losses occurring in North and South America (Grau et al., 2004). For example, in a particularly bad year for *Sclerotinia* on soybean in the United States in which it ranked second out of 23 diseases, an estimated cost of US$560 million was reported (Peltier et al., 2012). It has been estimated that, in bad years, losses in peanut yield can reach US$1 million in North Carolina alone (Smith et al., 2008).

Despite the regular application of fungicides, *Sclerotinia* diseases are one of the major causes of losses in horticultural crops (Vallalta and Porter, 2004; Saharan and Mehta, 2008). This includes both leafy vegetables, such as vegetable brassicas, and root vegetables, such as potatoes and carrots. The fungus can cause losses in both the field and under storage conditions, although most plants become infected in the field (Dillard, 1987). Lettuce is reported as one of the crops that are most susceptible to *Sclerotinia*. Losses in field-grown lettuce in the United Kingdom are commonly 10%, but greater losses of up to 50% can occur under wet conditions (Young et al., 2004). *S. Minor*-induced losses in lettuce are reported to range from 10 to 45% in intensive lettuce-growing regions of Australia, despite the use of fungicide in spray programs (Vallalta and Porter, 2004).

*Sclerotinia minor* is less common than *S. sclerotiorum* but has a somewhat narrower host range (Willetts and Wong, 1980). It is a major concern for peanuts as the cause of *Sclerotinia* blight (Chenault Chamberlin et al., 2010). Losses in peanut crops in North Carolina alone due to *Sclerotinia* blight have been estimated at US$1–4 million/year (Smith et al., 2008). Other crops impacted by *S. minor* include soybean and common beans (Grau et al., 2004).

Of the *Sclerotinia* species, *Sclerotinia trifoliorum* has the narrowest host range, infecting mostly legumes (Willetts and Wong, 1980). It causes white mold, crown, and stem rot of forage legumes, such as lucerne/alfalfa and clovers, and cool season grain legumes (e.g., chickpea, lentil, faba beans) (Jellis et al., 1990; Njambere et al., 2014). All species that are infected by *S. trifoliorum* are also susceptible to *S. sclerotiorum* (Willetts and Wong, 1980; Njambere et al., 2014).

## Pathogen Epidemiology and Disease Cycle

### *Sclerotinia* Disease Development and Symptoms

**FIGURE 1:**
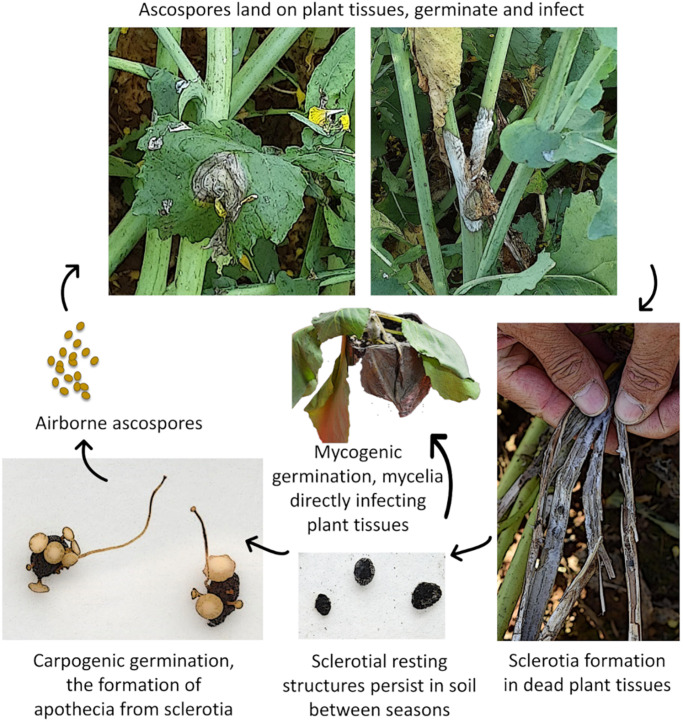
Generalised *Sclerotinia* disease cycle. Image credits: Smith, 2018 CSIRO and Tian, 2020 CSIRO.

As a necrotrophic fungal pathogen, the *Sclerotinia* fungus needs to kill plant cells in order to establish disease and obtain nutrients. It is such a destructive pathogen in agriculture, because it is able to infect plants at any growth stage including young seedlings, mature plants, and fruits in the field or in storage. In addition, when not infecting plants, the fungus may spend more than 90% of its life as sclerotia, its primary resting stage. Sclerotia are hard, melanised survival structures (Figure 1) that are resistant to desiccation and act as food storage reserves, allowing the fungus to survive in soil or stubble for up to 5 years or more (Agrios, 2005; Peltier et al., 2012; Young and Werner, 2012; Smolinska and Kowalska, 2018). These resting structures allow the pathogen to survive in the absence of a plant host and serve as a source of infection in subsequent crops (Agrios, 2005; Young and Werner, 2012). The sclerotia of *S. sclerotiorum* and *S. trifoliorum* are typically 2–5 mm in diameter but sometimes greater than 10 mm, while *S. minor* produces small sclerotia of 0.5–2 mm in diameter.

Both *Sclerotinia sclerotiorum* and *Sclerotinia trifoliorum* reproduce carpogenically by producing small (5–10 mm in diameter), tan, cup-shaped mushroom-like structures called apothecia (Figure 1) which contain sexual spores called ascospores (Willetts and Wong, 1980). Sclerotial germination and ascospore production are favoured by moist, cool soils with temperatures around 4–18°C; however, temperature requirements are variable depending on the origin of the *Sclerotinia* isolates (Willetts and Wong, 1980; Peltier et al., 2012; Smolinska and Kowalska, 2018). Cold-conditioned sclerotia within the top centimetres of soil typically begin to produce apothecia after 2–6 weeks at 15°C (Willetts and Wong, 1980; Young et al., 2004; Peltier et al., 2012; Clarkson et al., 2014). The apothecia-fruiting bodies burst, releasing thousands of ascospores that are readily spread by the wind throughout plant canopies and surrounding areas. *S. sclerotiorum* ascospores require certain environmental conditions in order to germinate and infect plants. Temperatures around 15–25°C and wetness or high humidity are required for >48 h (Young et al., 2004; Clarkson et al., 2014; Derbyshire and Denton-Giles, 2016; Willbur et al., 2019). Susceptible plant tissues such as flower petals and senescing leaves are typically infected first (Link and Johnson, 2007), serving as the nutrient source for *Sclerotinia* mycelium to grow and subsequently infect other plant tissues. In canola, for example, infected senescent petals are the predominant source of infection. They drop into the canopy and lodge onto leaf axils or stem branches, or stick to leaves and stems (reviewed in Derbyshire and Denton-Giles, 2016; Thatcher et al., 2017). Surveillance for infested petals is commonly performed to measure pathogen load in the field (reviewed in Derbyshire and Denton-Giles, 2016).

Infection can also occur myceliogenically by contact with hyphae from sclerotia onto plant tissues, or directly from contact with other diseased plants. This is a common infection route for *S. minor*, which rarely produces apothecia, but is relatively rare in *S. sclerotiorum* and *S. Trifoliorum* (Link and Johnson, 2007). The environmental drivers for differential carpogenic or mycelial germination of *S. sclerotiorum* or *S. trifoliorum* sclerotia are not well understood. A review of abiotic conditions promoting myceliogenic germination of *S. sclerotiorum* highlighted moisture availability and extremes in temperatures as key factors influencing myceliogenic germination (Lane et al., 2019).

Upon infection, typical *Sclerotinia* lesions first appear on the leaves and leaf axils as water-soaked spots or lesions with a pale greyish white or brownish white appearance caused by cell death and the action of pathogen pectolytic enzymes. The water-soaked lesions, particularly at nodes, can rapidly progress along and around the stem above and below the infected nodes. Infected leaves may also fall and lodge further down the canopy and spread the infection to other plants. Plants infested with *Sclerotinia* develop a white, cottony growth on the stems followed by the formation of sclerotia (Figure 1), which can occur inside or outside of stems, and pods of legumes such as soybean, or on canola pods if the weather is favourable (Fernando et al., 2004; Bolton et al., 2006; Kamal et al., 2016; Willbur et al., 2019). The infected part of the stem turns soft and greyish-white in colour. The lesions girdle the stem, cutting off vascular transport, causing infected stems to become bleached and stringy or shred, and plant tissues above the lesion wilt and die (Grau et al., 2004; Derbyshire and Denton-Giles, 2016; Willbur et al., 2019). Legume pods on infected plants may appear white in colour, smaller, and contain fewer seeds. They may also contain sclerotia. Other common symptoms include stunting, premature ripening, and lodging of plants (Willetts and Wong, 1980; Peltier et al., 2012). Sclerotia produced within infected stems can be distributed after harvest by equipment or may be harvested with the seed, causing contamination of seed lots. Therefore, it is important to screen seeds for contamination.

Infection by *Sclerotinia sclerotiorum* is often patchy across a field, and symptoms on row or broad acre grain and oilseed crops are generally only evident late in the season after the flowering stage (Grau et al., 2004; Derbyshire and Denton-Giles, 2016). Plants can be attacked at any growth stage, but infection by *S. sclerotiorum* generally occurs during flowering, because favourable weather conditions tend to coincide with a fully developed crop canopy supporting cool, moist, shaded conditions in soils, which encourage the development of apothecia and the release of ascospores (Willbur et al., 2019). Mist, dew, and fog are all potential sources of moisture. Disease development is dependent on sufficient moisture in the canopy and typically extended periods of leaf wetness. Losses tend to be greatest when cool (15–25°C), wet, humid conditions, combine with management practices that favour high yield potential such as high planting density, narrow row spacing, and high plant nutrition that support the development of dense canopies that can contain high humidity (Grau et al., 2004; Link and Johnson, 2007; Peltier et al., 2012). Crops grown by overhead spray irrigation will also favour disease development.

*Sclerotinia sclerotiorum* can infect vegetable crops at any growth stage. Infections typically occur on the stem or leaves at the base or from infection events on the top of densely grown crops (Dillard, 1987). Blossoms from weeds are a common source of nutrients for ascospores and, consequently, a source of fungal inoculum on nearby vegetable crops. Infection starts with water-soaked lesions that become infested with fungal mycelium. The host tissue becomes soft and watery, ultimately leading to complete crop failure, and sclerotes produced with the diseased tissues.

*Sclerotinia minor* infects through eruptive myceliogenic germination; therefore, infection below the canopy at roots, crowns, and leaves is observed. Disease symptoms of *S. minor* are similar to those of *S. sclerotiorum* but not necessarily coincide with flowering. Initially, water-soaked lesions appear on the stems or leaves. As the disease progresses, fluffy white mycelia may become visible, and then the lesions become bleached, and necrotic and infected stems become shredded and die (Smith et al., 2008).

Rots of forage legumes caused by *Sclerotinia trifoliorum* manifest as a soft rot of the crown and roots, which begins with the appearance of brown leaf lesions that then spread to stems and shoots. New growth on the infected plants begins to wilt, then dies. The dead tissues are often covered with white mycelium, particularly in wet, cool weather (Barbetti and You, 2014). While plants can be infected at all growth stages, losses tend to be most severe when infection occurs at the seedling stage (Kanbe et al., 2002).

### Pathogenicity

An understanding of the cytological and molecular mechanisms of *Sclerotinia* colonisation and infection has increased in recent years thanks to advances in genomic sequencing, transcriptomic analysis, and functional gene characterisation. Recent findings show that the interactions between *Sclerotinia* and its wide range of hosts may be significantly more sophisticated than previously assumed, with the molecular mechanisms of infection and pathogenicity recently well reviewed (Mbengue et al., 2016; Liang and Rollins, 2018; Xia et al., 2020). Here, we summarise these findings and provide an overview of *Sclerotinia* pathogenicity, and in subsequent sections, we will detail how this knowledge could be exploited in new controls for disease suppression.

#### Host Infection

As a canonical necrotrophic fungus, *Sclerotinia* attacks and kills the cells of its host by secreting an arsenal of cell wall-degrading enzymes and toxins, and it consumes dead cells for energy (Mbengue et al., 2016; Xu et al., 2018; McCaghey et al., 2019; Wang et al., 2019a). It has been proposed that this pathogen exhibits a dynamic two-phased infection model in which it first evades or counteracts host defence, colonising the host and growing in apoplastic spaces (Kabbage et al., 2015; Liang and Rollins, 2018; Ding et al., 2021). Through the production of cell-degrading enzymes and toxins, the pathogen then progressively kills and degrades the host cells. It is further proposed that this phased infection process is not just defined at the temporal or spatial level, but rather the possibility that it isdefined through different sectors or developmental stages of the advancing fungal colony (Kabbage et al., 2015).

The initial colonisation of the host by *Sclerotinia* occurs *via* a compound appressoria (a modified hyphal tip), which penetrates the cuticle of the host *via* a penetration peg. Following initial penetration, the colonisation phase involves the growth and branching of sub-cuticular hyphae (Liang and Rollins, 2018). There is evidence that these subcuticular hyphae can spread several cell layers ahead of killed epidermal cells, in which the hyphae are interacting with healthy cells (Kabbage et al., 2015). Once the subcuticular hyphae are established, they branch into smaller, ramifying hyphae that grow inter- and intra-cellularly into the epidermal and mesophyll cells. Liang and Rollins (2018) noted that subcuticular and ramifying hyphae have distinct morphology and colonisation patterns, suggesting potential functional specialisation, and that subcuticular hyphae are likely to be more important in defence suppression and infection establishment, while ramifying hyphae are more important in initiating cell death and cell wall degradation.

Further evidence for the two-phase infection model in *Sclerotinia* was found in a study analysing sequential gene expression patterns during infection of *Brassica napus* (Seifbarghi et al., 2017). During the first 12 to 24 h post-infection period the expression of *Sclerotinia* effector-like genes (e.g., genes encoding the LysM domain protein and a cysteine rich protein) associated with suppression of the pathogen recognition and defence systems in the host was upregulated. At the later stage of infection (after 24 h) *Sclerotinia* genes associated with the induction of necrosis and programmed cell death were upregulated.

The secretion of a wide array of cell-wall-degrading enzymes facilitates the degradation of host cell walls, as well as numerous proteases and hydrolases, to macerate tissues and release nutrient sources (Mbengue et al., 2016; Xu et al., 2018; McCaghey et al., 2019; Wang et al., 2019a). These include proteinases, cutinase, cellulases, polygalacturonases, glucanases, and xylanases. It has been suggested that the diversity of enzymes produced by *Sclerotinia* may enhance its adaptability and therefore contribute to its wide host range (Ding et al., 2021).

#### Secreted Proteins and Host Defence Suppression

Important virulence components of *Sclerotinia* include secreted and effector-like proteins and are reviewed in detail by Derbyshire et al. (2017), Xu et al. (2018), McCaghey et al. (2019), Xia et al. (2020), and Shao et al. (2021). Historically, fungal effectors have been defined as small, secreted proteins that modulate the host cell to facilitate infection. More broadly, they encompass a wider array of proteins or molecules that function to establish and progress disease.

Bioinformatics approaches applied to the full genome sequence of *S. sclerotiorum* have identified a large number of genes encoding a range of virulence-related secretory effector proteins (Amselem et al., 2011). Secretome analyses have shown that *S. sclerotiorum* has the potential to produce 400 secreted proteins including nearly 80 virulence factor candidates (Guyon et al., 2014; Heard et al., 2015; Derbyshire et al., 2017). Of those characterised to date, manipulation of host salicylic acid, jasmonic acid, and ROS signalling are common themes to suppress or interfere with host defense responses (McCaghey et al., 2019; Wang et al., 2019a; Ding et al., 2021). For example, the *S. sclerotiorum-*secreted integrin-like protein SsITL suppresses host immunity at the early stage of infection (Mbengue et al., 2016; Tang et al., 2020). It interacts with the plant chloroplast-localised CAS (chloroplast-localised calcium-sensing receptor) protein that is a positive regulator of salicylic acid signalling and resistance against *Sclerotinia*. Manipulators of host cell death include the *Sclerotinia* Cu/Zn superoxide dismutase SsSOD1 and the Ca-binding SsCAF1 protein also involved in *Sclerotinia* development (reviewed in McCaghey et al., 2019; Xia et al., 2020). The chorismate mutase enzyme Ss-Cmu1 is predicted to be secreted and dampen host salicylic acid synthesis (Liang and Rollins, 2018). In another example, the *S. sclerotiorum* small, cysteine-rich secreted protein SsSSVP1 induces plant cell death and interacts with the plant QCR8, a subunit of the cytochrome b-c1 complex of mitochondrial respiratory chain, and disturbs the localisation of QCR8 in mitochondria (Lyu et al., 2016). Another strategy employed by *S. sclerotiorum* has been recently shown in the *Sclerotinia*-Brassica pathosystem. Chen et al. (2020) demonstrated that *S. sclerotiorum* was able to metabolise, *via* a hydrolase, toxic isothiocyanates produced by the plant and therefore was able to promote its growth and contribute to its virulence on glucosinolate-producing plants. Together, these examples highlight the multiple strategies employed by the *Sclerotinia* secretome to manipulate or avoid host cell processes and likely contribute to its broad host range and effectiveness as a destructive plant pathogen. Further functional characterisation of these and other *Sclerotinia* virulence-related secreted proteins may provide new targets for disease control targeting either the pathogen directly or through the modification of its host target.

#### The Roles of Oxalic Acid, ROS, and Host Cell Death

*Sclerotinia* also produces the non-selective phytotoxin, oxalic acid, that contributes to pathogenesis through acidification and suppression of host defence responses by manipulating the host redox environment (Williams et al., 2011; Smolinska and Kowalska, 2018). Evidence for the involvement of oxalic acid in plant infection has been demonstrated by the recovery of oxalate from infected tissues (Bateman and Beer, 1965; Maxwell and Lumsden, 1970; Marciano et al., 1983; Godoy et al., 1990) or by injection of oxalate into plants followed by the development of *Sclerotinia* disease-like symptoms (Bateman and Beer, 1965; Noyes and Hancock, 1981). In addition, it could be demonstrated that *Sclerotinia* mutant strains that were deficient in oxalate synthesis were non-pathogenic but regained normal virulence upon regaining their oxalate synthesis capacities (Godoy et al., 1990). Recent studies proposed that it is not oxalic acid specifically that is necessary for the ability of *Sclerotinia* to cause disease but rather its acidic pH (reviewed in Xu et al., 2018).

In neutral or alkaline environments, *Sclerotinia* grows slowly and produces acids such as oxalic acid to acidify its environment (Tourneau, 1979). The fungus increases its growth rate under acidic conditions (Maxwell and Lumsden, 1970) and initiates the production of sclerotia (Rollins and Dickman, 2001; Chen et al., 2004). Lytic and hydrolytic enzymes are expressed and secreted under acidic conditions, which are needed for optimal enzyme activity and are necessary for disease development (Cotton et al., 2003; Girard et al., 2004; Kasza et al., 2004; Li et al., 2004; Seifbarghi et al., 2017). If the ambient pH gets too low, *Sclerotinia* will be able to produce ammonia or oxalate decarboxylase that degrades oxalate, thereby raising the pH (Vega et al., 1970; Magro et al., 1988; Billon-Grand et al., 2012; Liang et al., 2015). A recent study compared the pathogenicity of different *Sclerotinia* isolates in canola and observed a correlation between pathogen aggressiveness and its ability to acidify its environment (Denton-Giles et al., 2018). The ability to sense and change pH in its environment enables *Sclerotinia* to survive under many different conditions and thereby makes it one of the most successful pathogens of plants.

In addition to its direct effect on cellular pH, oxalic acid has been shown to induce programmed cell death, and it is thought that the manipulation of host programmed cell death plays a crucial role in the pathogenicity of *Sclerotinia* (Kim et al., 2008; Mbengue et al., 2016). Oxalic acid induces increased levels of ROS in the host, which is also associated with programmed cell death. When ROS production is inhibited, apoptotic-like cell death due to oxalic acid is also inhibited (Kim et al., 2008). Kim et al. (2008) suggested that additional roles of oxalic acid include acting as a signalling molecule that induces programmed cell death. *Sclerotinia* also produces a range of other factors that induce the necrosis of host cells. Most are proteins or peptides such as ethylene-inducing peptides, endopolygalacturonases, and a cutinase (Liang and Rollins, 2018). Two small secretory proteins, Ss-SSVP1 and Ss-CP1, have been shown to induce cell death (Lyu et al., 2016; Yang et al., 2018).

#### Gene Silencing and sRNAs

A recent study by Derbyshire et al. (2019) showed that during infection, *Sclerotinia* produces at least 374 distinct small RNAs, many of which downregulate functional domains associated with plant immunity. These small RNAs induce gene silencing through RNA interference (RNAi) pathways (Mbengue et al., 2016).

A genome wide association (GWAS) study has shown that bioinformatically predicted targets of *Sclerotinia* small RNAs included functional domains associated with plant immunity and quantitative disease resistance (Derbyshire et al., 2019). Mutants of both plant and fungal RNAi components were found to have reduced silencing of host immunity genes and therefore reduced disease symptoms (Weiberg et al., 2013).

### Foe to Most, Friend to Some?

While *Sclerotinia sclerotiorum* predominantly infects dicots, in a recent study, it was found to grow endophytically within several monocots including wheat, barley, oat, rice and maize (Tian et al., 2020). While it is not uncommon for fungal pathogens of a plant species to colonise another plant species and not cause disease, in the study by Tian, they found that *S. sclerotiorum* colonisation actually had a positive influence on the host. Endophytic growth within wheat reduced disease severity incited by the phytopathogenic fungal pathogens that cause *Fusarium* head blight and stripe rust, likely through manipulation of defence responses and hormone signalling. It is interesting that such a broad host range and highly destructive pathogen of dicots had a beneficial effect on monocots. These results have implications in the cultural management of *Sclerotinia* in farming systems where susceptible break crops such as canola and legumes are grown in rotation or in close proximity to cereals. Further studies are needed to determine if mycelium carry-over between seasons on monocot stubble can serve as an inoculum source for the following year.

## Disease Control

**FIGURE 2:**
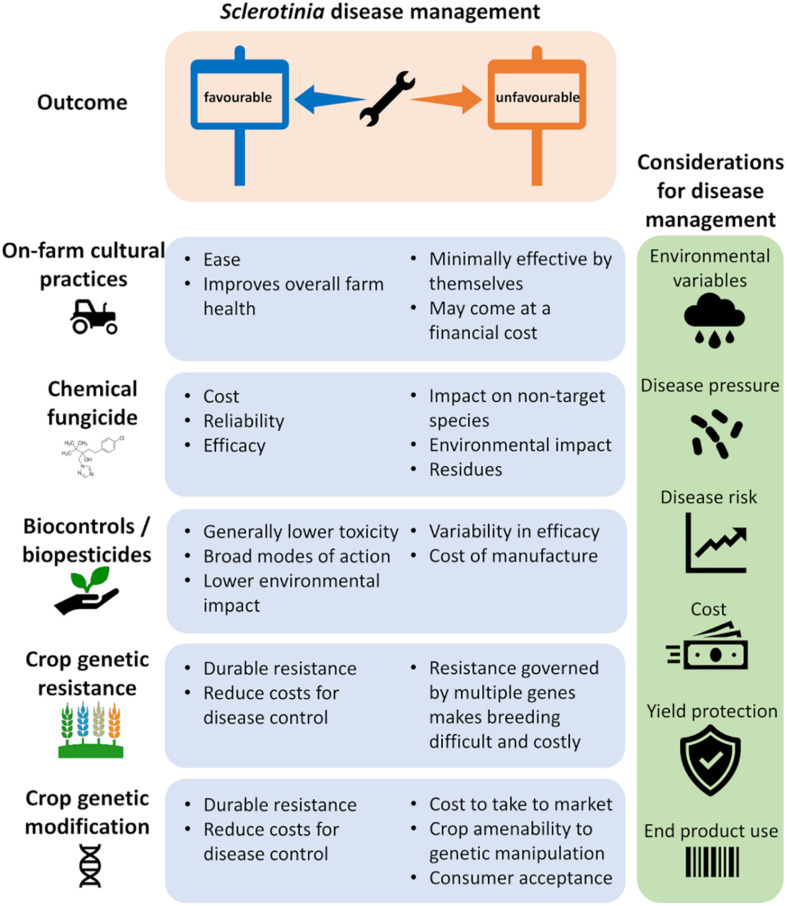
*Sclerotinia* disease management. Current strategies to control *Sclerotinia* diseases rely on integrated disease management, each with its pros and cons (blue panels) toward the desired outcome. This may involve a combination of management strategies suited to the host crop and requires consideration and monitoring of multiple factors to guide decision on best suited disease control tools (green panel).

As with many diseases, there is no single treatment that can completely control *Sclerotinia*, so most growers employ an integrated management approach to reduce their risk of losses from disease. This involves combining several approaches including cultural practices, variety selection, and chemical and biological controls (Peltier et al., 2012). With limited genetically resistant cultivars available to growers and the broad host range of the pathogen, cultural and management practices, such as fungicide use, are the predominant approaches for control but provide variable protection depending on the timing of application. Growers, therefore, need to consider environmental variables, disease pressure, and risks when planning their management strategy (Figure 2).

### Cultural Practices

Cultural and agronomic measures are an important part of disease management and can reduce disease severity but are not effective by themselves in controlling the disease. Cultural practices include crop rotation, reducing plant density, and practices to reduce ascospore production and release. Because *Sclerotinia* has a wide host range, many broad leaf crops and weeds support the disease, so weed management and careful selection of crops to rotate are required, as repeated cropping of susceptible crops increases sclerotial numbers in each subsequent crop. Small grain crops (maize, wheat, barley, oats, sorghum) are not susceptible to infection by *Sclerotinia* spp. and so are suitable rotation crops but a break of 2–3 years may be required to decrease the number of sclerotia in soil (Peltier et al., 2012). However, in light of the potential for these small grain crops to still be colonised by *Sclerotinia* (Tian et al., 2020), avoidance of infested or adjacent fields for 1–4 years could be considered the best management option if economically viable.

Canopy management, including wider row spacing or lower seeding rates, can be used to increase air flow and decrease humidity in the crop canopy. Studies on a range of different crops have shown that this can reduce numbers of apothecia and disease incidence (McDonald et al., 2013). Reducing fertilisers and delaying planting to manage vegetative growth should also be considered, as over-fertilising and early planting can result in tall, bulky crops at the time of flowering that will create denser canopies, and, with high rainfall, will put crops at higher risk of disease.

Stubble management practices to minimise the carry-over of viable sclerotia between seasons have demonstrated efficacy. These include burning crop residues or using irrigation to increase rotting of sclerotia. Other studies have suggested that there may be lower disease incidence in no-till systems (Kurle et al., 2001; Simpfendorfer et al., 2004) but these results are inconsistent (Peltier et al., 2012). It is speculated that sclerotia may be more likely to degrade in no-till systems because they are not buried and are more prone to attack by predators, desiccation, or exposure to UV in the upper soil layers (Peltier et al., 2012). It is also advisable not to keep seeds from infected crops for replanting, as this will also increase the risk of spreading infection into potentially previously clean areas.

### Crop Resistance

For all crops that are susceptible to *Sclerotinia* species, it is generally advised that where partially resistant cultivars are available, they should be used as part of an integrated management approach to reduce the risk of yield losses and to decrease the load of inoculum in future seasons (Kurle et al., 2001; Grau et al., 2004; Smith et al., 2008).

There are ongoing efforts to breed *Sclerotinia*-resistant cultivars of a range of broad acre and horticultural crops (Kim and Diers, 2000; Arahana et al., 2001; Cruickshank et al., 2002; Lithourgidis et al., 2005; Vuong et al., 2008; Chenault Chamberlin et al., 2010; Barbetti and You, 2014; Taylor et al., 2015; Derbyshire and Denton-Giles, 2016; Bennett et al., 2018). In all cases, it is important to consider that the disease screening method is reflective of conditions experienced by crops grown under field conditions. For example, in canola, one of the most significant limitations in high throughput seedling assays is that plant disease resistance is not measured at the growth stage during which the disease is most likely to occur in the field, which means that plants should be grown at the flowering developmental stage (Derbyshire and Denton-Giles, 2016; Thatcher et al., 2017).

Molecular breeding is an important strategy in several crops for improving host resistance as an avenue for disease control. There is no genetic source of complete plant resistance to these broad-host range pathogens known to date, but partial resistance has been identified in several economically important crops (e.g., canola and soybean) (Mbengue et al., 2016; McCaghey et al., 2019). Resistance to *Sclerotinia* is polygenetic and made up of the complex interaction of numerous minor effect genes (Wang et al., 2019a). This results in quantitative disease resistance, in which a natural population of plants will display a continuum of resistance phenotypes from highly susceptible to partially resistant.

The current status of resistance mapping and breeding is well reviewed by Wang et al. (2019a) and Ding et al. (2021). Early studies screening for resistance loci in *B. napus* used quantitative trait loci (QTL) mapping. More recently, alternative approaches that include genome-wide association mapping studies using natural plant populations have been applied. This, paired with high-throughput sequencing, omics, and improved data analytics combined with an increased understanding of molecular mechanisms of plant defense against *Sclerotinia* have improved the identification of quantitative resistance gene/loci and of host genes or processes that can be targeted and manipulated for enhancing resistance or reducing susceptibility. For example, Ding et al. (2021) broadly categorised resistance responses into three types: (1) the activation of defence signaling pathways, (2) the production of secondary metabolites that kill *Sclerotinia* or inhibit growth and infection processes, and (3) the production of activating enzymes, proteins, or antimicrobial peptides that affect *Sclerotinia* cell wall integrity or block its pathogenicity factors. Wang et al. (2019a) noted that the GWAS studies on *Sclerotinia* resistance in *B. napus* predominantly linked loci-containing genes involved in downstream defence responses, ROS production, detoxification, oxidative protection, and secondary metabolite enzymes. These findings suggest that *Sclerotinia* resistance is likely a function of a variety of cellular processes and that this needs to be taken into account when breeding for crop varieties with enhanced resistance.

#### Natural Sources of Resistance or Non-GM Approaches

The fact that *Sclerotinia* tolerance in several species is controlled by multiple minor genes makes breeding resistant varieties difficult and costly, and raises the risk that undesirable traits can be inadvertently introgressed through the process of linkage drag (Wang et al., 2019a). In some cases, QTLs that are associated with physiological resistance to *S. sclerotiorum* have been reported (Grau et al., 2004). It is thought that the severity of disease caused by *S. sclerotiorum* is governed by a combination of physiological resistance (e.g., phytoalexin production) and disease escape mechanisms (e.g., open canopy architecture, lodging resistance, and timing of maturity) (Grau et al., 2004). This further complicates efforts to breed *Sclerotinia*-resistant varieties, as the disease tolerance phenotypes may not be suitable as breeding material.

There is a relatively large body of information on quantitative trait locus mapping data available for resistance to *Sclerotinia* in various hosts, but relatively little is known about the molecular basis for the quantitative trait loci. The advances in molecular breeding are likely to be made possible because of the work being done to increase the understanding of the molecular basis of host genetic resistance (Wang et al., 2019a). Using a GWAS approach analysing global B. napus germplasm, Gyawali et al. (2016) identified 669 polymorphic loci that were associated with Sclerotinia resistance including 21 alleles related to resistance and 13 related to susceptibility. A map of *Sclerotinia* resistance has been constructed in *B. napus* using 347 markers and integrated 35 QTLs that have been linked to disease resistance (Li et al., 2015). Similar studies in soybean have identified 103 QTLs that have been mapped to 16 and 11 loci that were significantly correlated with resistance in field and greenhouse by GWAS (Wen et al., 2018). It has been noted in both canola and soybean that resistance to *Sclerotinia* is conferred by minor QTLs, but each only makes a small contribution (typically less than 10%) to phenotypic variance (Wen et al., 2018; McCaghey et al., 2019).

In canola, the lack of complete genetic resistance has prompted researchers to screen close relatives of *Brassica napus* such as *Brassica oleracea* and also wild crucifers that lie outside the *Brassica* genus (reviewed in Derbyshire and Denton-Giles, 2016). Partial stem resistance has been identified through a variety of screening methods (Garg et al., 2008, 2010; Denton-Giles et al., 2018). The challenge will be to breed this material into elite cultivars. Avoiding petal infection is another option that has been explored. Apetalous *B. napus* plants develop less disease but are still equally infected by *S. sclerotiorum* as full-petalled cultivars (Young and Werner, 2012).

In soybean, there are no cultivars with complete resistance to *Sclerotinia sclerotiorum*, but there are partially resistant varieties available (Grau et al., 2004; McCaghey et al., 2019; Willbur et al., 2019). There are commercial varieties of peanut available that are resistant to *S. minor* and *S. sclerotiorum*. It has been estimated that the resistant peanut cultivars, Toalson, Tamspan 90, and Tamrun 98, save the United States peanut industry from ∼US$5 million in losses due to *Sclerotinia* annually (Bennett et al., 2018). There are ongoing efforts to breed elite varieties with *S. sclerotiorum* and *S. minor* resistance that are adapted to a wider range of environments (Cruickshank et al., 2002). There is evidence that resistance to *S. trifoliorum* exists within several forage and grain legumes such as alfalfa, clover, and faba beans, but there are currently no commercially available resistant cultivars, and breeding efforts are ongoing (Halimi et al., 1998; Kanbe et al., 2002; Lithourgidis et al., 2005; Mikaliuniene et al., 2015). Screening for *S. sclerotiorum* resistance in wild *Cicer* populations, with the aim to adopt this into commercial chickpea varieties, identified partial stem resistance (Mwape et al., 2021).

#### Genetic Modification

**FIGURE 3:**
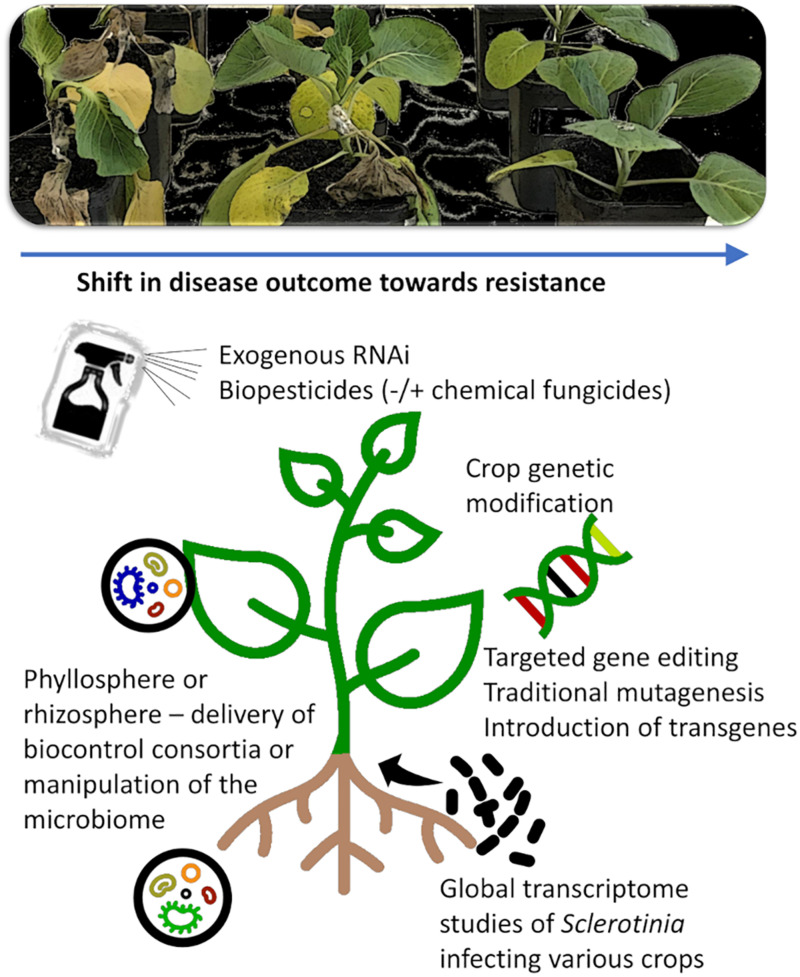
New opportunities and tools for *Sclerotinia* control. New tools exist or are emerging that allow targeted manipulation of pathogen, host, or beneficial microbial populations that facilitate a reduction in *Sclerotinia* disease symptom development. These includes exogenous controls such as RNAi or biochemicals and biopesticides that can act directly on the pathogen (e.g., antifungal activity, fungal development) or indirectly by enhancing plant defense responses. Powerful endogenous controls such as crop genetic modification can provide durable resistance, but the process is costly, and not all crops are readily transformable or afford public acceptance. New knowledge of the pathogenicity and virulence of *Sclerotinia* species can be acquired through global omics studies on the pathogen infecting diverse crops of varying disease resistance, and this knowledge can be used to identify essential pathogen processes or weaknesses that can be targeted in new management tools.

The lack of strong, major effect resistance in most crops makes classical breeding a challenging task. In crops where genetically modified (GM) varieties are already available for other traits, such as canola or soybean, transgenic or targeted genetic modification approaches for *Sclerotinia* resistance may be an economical option (Figure 3).

The overexpression of oxalic acid degrading oxalate oxidase genes in a number of crops has enhanced resistance to *S. Sclerotiorum* (Lithourgidis et al., 2005; Yang et al., 2019; van Esse et al., 2020). In most cases, complete resistance is not observed but rather a delay in disease progression. This may, however, be sufficient in the field to provide a yield benefit or reduce the requirement for fungicide applications.

A general strategy to enhance plant resistance to pathogens is to upregulate the expression of key or master switches of defence responses such as transcription factors or mitogen-activated protein kinases (MPKs) (Thatcher et al., 2005; Amorim et al., 2017). The overexpression of specific WRKY transcription factors or MPKs in Arabidopsis and/or canola has been demonstrated to provide increased resistance against *Sclerotinia* (Chen et al., 2013; Liu et al., 2018; Wang et al., 2019b). Other approaches taken include the overexpression of downstream pathogen inhibiting defence genes, such as chitinase genes or polygalacturonase-inhibiting proteins, to slow down disease progression (Zarinpanjeh et al., 2016; Wang et al., 2018; Yang et al., 2020). More recently, plant-derived antifungal metabolites have shown a potential to inhibit *Sclerotinia*, and their expression could be manipulated for enhanced resistance. For example, Ranjan et al. (2019) demonstrated that metabolite extracts from the stems of enhanced resistance soybean lines had an antifungal activity and targeted pathogen ergosterol biosynthesis, disrupting enzymes involved in fungal lipid and sterol biosynthesis.

More recently, targeted gene editing of a *Sclerotinia* effector host target has shown potential as a new strategy to deliver increased resistance. In the study by Zhang et al. (2021), they found that *Brassica napus* has eight homologous copies of BnQCR8, the plant target of the *Sclerotinia* effector-like protein SsSSVP1 (Lyu et al., 2016). Using CRISPR/Cas9 to reduce the BnQCR8 copy number, they found that *B. napus* mutants with one or more edited copies displayed stronger resistance against *S. sclerotiorum*. The mutants also showed increased resistance to the related necrotrophic fungal pathogen *Botrytis cinerea*, which contains an SsSSVP1 homologue. This study highlights the power of editing host targets shared by pathogen effectors.

Numerous other genes have been implied in resistance responses against *Sclerotinia*, highlighting the arsenal of attack strategies employed by this pathogen over a diverse array of hosts (reviewed in Wang et al., 2019a). The challenge, therefore, if a targeted genetic manipulation approach is taken, is what host gene(s) will be the best targets, or, if the transgenic approach is undertaken, what transgenes will be the most effective. This may include the expression of antimicrobial proteins, the overexpression of specific defence pathways or key regulators, or employing RNA interference (RNAi) approaches to target key host or *Sclerotinia* processes (Andrade et al., 2016; McCaghey et al., 2019; van Esse et al., 2020; Xia et al., 2020).

### Chemical Fungicides

In addition to cultural control, fungicides are widely used to control *Sclerotinia*. A range of fungicides are commercially available to prevent disease losses; however, the environmental drivers of disease can make the timing of application decisions difficult. The decision to apply a fungicide should also not only be made with the current harvest in mind but also aimed at reducing sclerotia load in the soil for future crops. The decision of whether to apply fungicides often becomes an economic trade-off between the cost of fungicide applications and the risk of disease (Figure 2).

Several different classes of fungicides with diverse modes of action are used globally to treat *Sclerotinia* on different crops. This includes anilinopyrimidines (inhibit methionine biosynthesis), methyl benzimidazole carbamates (MBCs) (inhibit cell division by disrupting microtubule formation), dicarboxamides (thought to inhibit osmotic signal transduction), demethylation inhibitors (DMIs) (inhibit membrane sterol biosynthesis and the development of functional cell walls), quinone outside Inhibitors (QoIs) or strobilurins (inhibit mitochondrial respiration), and succinate dehydrogenase inhibitors (SDHIs) (inhibit mitochondrial respiration) (reviewed in Peltier et al., 2012; Derbyshire and Denton-Giles, 2016). Not all are registered for use on all crops, and in some cases there are restrictions on the number of applications of certain fungicides per crop, per season. Several chemical classes have limited systemic movement in the plant, meaning they are not functional on non-treated plant tissues (Peltier et al., 2012).

In broad acre or flowering row crops such as canola or soybean, fungicides can reduce disease incidence, but the timing of application is critical and can be difficult to achieve. Plants typically become susceptible to infection once flowering commences. In most years, fungicides are targeted at the early flowering stage, but optimal timing varies depending on the season and is only economical when there is a moderate to high risk for *Sclerotinia* infection (Mueller et al., 2002; Peltier et al., 2012; Derbyshire and Denton-Giles, 2016; Willbur et al., 2019). The efficacy of fungicides greatly decreases if they are applied after symptoms become visible. Foliar fungicides applied during early flowering can provide some protection but are typically preventative rather than curative, and the decision to apply must be made early, before symptoms have developed (Peltier et al., 2012). The goals of fungicide application are to protect early petals, achieve maximum penetration of the product into the crop canopy, and protect potential infection sites. For regions with high disease levels or long-flowering periods, a second spray is often targeted at later flowering stages (Paulitz et al., 2015). Fungicide application during flowering constitutes a significant monetary investment, and because of the sporadic nature of the disease (variability in disease incidence from year-to-year, region-to-region, and field-to-field), routine prophylactic application of fungicides is uneconomical and undesirable. The long flowering time of canola in some growing regions such as Australia (up to 6 weeks) and the effective activity of preventative fungicides (up to 3 weeks) often necessitate more than one fungicide application. In some instances, high infection levels are observed in canola even after two foliar fungicide applications. This indicates the difficulty in controlling the disease and the importance of understanding the environmental triggers for more targeted controls.

Fungicides recommended for control of *S. minor* on peanut, for example, include aminopyridines, dicarboxamides, and SDHIs (Ryley et al., 2000; Smith et al., 2008). All the three compounds are considered preventive and therefore must be applied before the onset of disease. As with the control of *S. sclerotiorum*, several fungicide applications may be needed to provide continuous protection in years with conditions highly favourable to disease development (Ryley et al., 2000). For many growers, this is not economical, and most will make a maximum of two applications (Smith et al., 2008).

Several fungicides have been trialled for their ability to protect against *Sclerotinia trifoliorum* in pasture legumes but, similar to treatments for *Sclerotinia sclerotiorum* and *Sclerotinia minor*, they are often not considered cost-effective (Kandula et al., 2015). Controlling *S. trifoliorum* in alfalfa is problematic. Several studies have suggested that it can be controlled with monthly sprays of dicarboxamides (Hoveland et al., 1996; Sulc and Rhodes, 1997). More recently Frate and Long (2005) found that dicarboxamides reduced disease symptoms and increased biomass yields of alfalfa in fields with known disease risk.

In leafy vegetable crops such as lettuce, the accurate timing of fungicide application is needed for efficacious control. This has prompted the development of disease forecasting models (Clarkson et al., 2014). Strategic plant-targeted sprays of dicarboxamides can be effective in *Sclerotinia* control, resulting in 80–96% reduction in *Sclerotinia* lettuce drop disease (Vallalta and Porter, 2004). Drenching of plants at transplanting and fungicide applications at the early crop stage before canopy closure are critical for controlling mycelial growth and preventing lower leaf infections. However, in some countries, permitted fungicide options are limited, and the increasing pressure to produce vegetable crops with low pesticide inputs necessitates alternate options to be implemented. In lettuce, most of the other registered fungicide classes (MBCs, strobilurins, aminopyridines, DMIs) were found to be less effective than dicarboxamides under high disease pressure (Pung and O’Brien, 2001; Vallalta and Porter, 2004).

Because most major fungicide classes rely on a single mode of action, the chance for the development of resistance in pathogen populations might be considered high (Derbyshire and Denton-Giles, 2016). However, *Sclerotinia* appears to have a low propensity for resistance, as it is considered homothallic (the ability of a single spore to produce a sexually reproducing colony) or undergoes asexual reproduction (Aldrich-Wolfe et al., 2015). However, instances of resistance to MBCs and dicarboxamides have been reported globally, and strains with decreased sensitivity to SDHIs have been reported in France (Ma et al., 2009; Wang et al., 2015; Derbyshire and Denton-Giles, 2016; reviewed in Liu et al., 2021). It is, therefore, important to not rely on a single mode of action to control *Sclerotinia*. Furthermore, the selective pressure caused by the overuse of fungicides may create resistance in other non-target pathogen populations. Recently, Liu et al. (2021) assessed the DMI fungicide metconazole for its potential usefulness in *Sclerotinia* control. They found no cross-resistance between metconazole and representative fungicides of the MBC, dicarboxamide, and SDHI classes over the 22 *S. sclerotiorum* isolates they tested, concluding that metconazole, if registered, could be used in spray rotations.

### Biological Control, Biopesticides, and Microbiome Manipulation

The high risk of losses as well as difficulty controlling *Sclerotinia* using traditional methods has driven research into alternative approaches. There is a significant body of research into potential biocontrols for *Sclerotinia* spp. Several different species of fungi, such as *Coniothyriumminitans* and *Trichoderma* spp., and bacteria, such as *Pseudomonas fluorescens*, *Bacillus* spp., and *Streptomyces* spp. are known to be antagonistic to *Sclerotinia* spp. (Whipps and Gerlagh, 1992; De La Fuente et al., 2004; Gorgen et al., 2009; Luduena et al., 2012; Zeng et al., 2012b; Chen et al., 2016b; Smolinska and Kowalska, 2018). Importantly, biocontrols offer new modes of action and can be interchanged with chemical fungicides, thereby reducing the reliance on chemicals whilst also aiding to prolong chemical modes of action and reducing the development of resistant pathogen populations (Figure 2).

While many studies show the antagonism of *Sclerotinia* spp. *in vitro* with a range of fungi and bacteria, the efficacy needs to be proven *in-planta* and, critically, in field trials where performance is strongly influenced by environmental factors (Boland, 1997). Many biocontrol candidates that appear promising in the lab fail to show sufficient efficacy in the field, as their efficacy can be highly influenced by environmental conditions (Chitrampalam et al., 2008). The key to their success will depend on correct delivery and ensuring durability under multiple environments. This was clearly articulated in a study of a commercial product for the biocontrol of *S. sclerotinia*. Nicot et al. (2019) found differences in the susceptibly of 75 *S. sclerotiorum* isolates, highlighting the need to test biocontrols against pathogen species diversity panels and the possibility of biocontrol selection pressure on pathogen populations, as commercial biocontrol products become more widely adopted by growers. For *Sclerotinia* control, several different biocontrol species have been commercialised, and there are ongoing efforts to develop other promising candidates. Several commercial examples are discussed below.

#### Beneficial Fungi

Of the range of potential biocontrol agents for *Sclerotinia*, the fungal parasite *Coniothyrium minitans* is the best studied and commercially established. *C. minitans* was first isolated from infected sclerotia of *S. sclerotiorum* in 1947 (Tribe, 1957). The fungal predator attacks and degrades sclerotia in the soil (de Vrije et al., 2001). The commercially available biocontrol formulation of *C. minitans* is Contans WG (Bayer CropScience, Cambridge, United Kingdom). Many other *Coniothyrium* spp. are necrotrophic plant pathogens, but *C. minitans* appears to have lost the ability to infect plants and become specialised in the infection of sclerotia in the soil as food source (Whipps and Gerlagh, 1992). Sclerotia attacked by *C. minitans* will not produce apothecia and, therefore, will not produce ascospores that initiate the onset of disease symptoms (de Vrije et al., 2001). The efficacy of *C. minitans* in controlling *Sclerotinia* disease has mostly been shown on high value crops such as canola, sunflower, lettuce, cucumber, beans, and peanuts (de Vrije et al., 2001; Jones and Whipps, 2002; Chitrampalam et al., 2008; McQuilken and Chalton, 2009; Ojaghian, 2010). In field trials of *C. minitans* to control *S. sclerotiorum* across multiple crops, reductions of 10–70% in disease symptoms have been reported (Whipps and Gerlagh, 1992; Zeng et al., 2012a; reviewed in Derbyshire and Denton-Giles, 2016; Smolinska and Kowalska, 2018). Reductions in sclerotia development by as much as 95% have also been reported [soybean (Zeng et al., 2012a)]. Glasshouse trials for the protective effect of Contans WG in combination with low doses of a range of fungicides on bean (*Phaseolus vulgaris*, L.) showed that Contans WG applied with a low dose of a dicarboximide fungicide completely eliminated the formation of symptoms in infected plants (Elsheshtawi et al., 2017), demonstrating the potential role of Contans WG in an integrated pest management system.

There is sufficient evidence supporting the efficacy of *C. minitans* in supressing *Sclerotinia* spp. that the commercially available formulation of Contans WG is recommended as part of an integrated pest management approach in some parts of the world. It can be applied as a soil drench or applied to heavily diseased crop debris post-harvest before they are ploughed into the soil to destroy sclerotia and reduce the risk of infestation in future crops (de Vrije et al., 2001; Yang et al., 2010). Despite several reports of *C. minitans* suppressing *Sclerotinia*, its efficacy in the field is also reported as inconsistent and may be linked to varying susceptibility of different *Sclerotinia* strains (reviewed in Derbyshire and Denton-Giles, 2016; Kamal et al., 2016; Nicot et al., 2019).

Another fungal biocontrol example is the use of *Trichoderma* strains where many studies have shown that several *Trichoderma* spp. interfere with hyphal growth of *Sclerotinia* spp., parasitise sclerotia, and reduce apothecia abundance in laboratory studies (Woo et al., 2014). There are over 100 commercially registered biofungicide and/or growth-promoting products based on various *Trichoderma* spp. (reviewed in Woo et al., 2014), and there is still a considerable research activity in biocontrol using *Trichoderma* spp. Reports on *Sclerotinia* field efficacy are, however, limited (Smolinska and Kowalska, 2018). Positive examples include disease control on cabbage and legumes with reductions in sclerotia levels and/or up to a 50–65% decrease in disease symptoms (Zeng et al., 2012a; Geraldine et al., 2013; Jones et al., 2014).

#### Beneficial Bacteria

It is known that several different species of bacteria, many commonly isolated from healthy plant tissues, rhizospheres or bulk soils, suppress pathogens and promote plant growth. Most of these bacteria directly antagonise pathogens through the production of antimicrobial compounds or lytic enzymes, directly out-compete pathogens or sequester nutrients from them, or induce systemic disease resistance within the plant, helping it to fight off pathogen attacks. Some beneficial bacteria also promote plant fitness by producing plant hormone-mimics and antioxidant compounds (Berg and Hallmann, 2006; Narayanasamy, 2013; Rey and Dumas, 2017).

There are numerous studies demonstrating the suppression of *Sclerotinia* by *Bacillus in vitro* and exploring the mechanism of suppression (Li et al., 2009; Tonelli et al., 2010; Luduena et al., 2012; Figueredo et al., 2014; Vinodkumar et al., 2017; Ansary et al., 2018; Lopes et al., 2018; Massawe et al., 2018). The efficacy of Bacillus spp. in preventing and treating *Sclerotinia* disease *in planta* and in the field is not as well demonstrated. Examples of field evaluation include *B. subtilis* (BY2) and *B. megaterium* (A6) protecting canola in field studies, leading to statistically significant increases in grain yield (Hu et al., 2013, 2014). In three field trials run over two seasons, Kamal et al. (2015) showed the protection of canola against *S. sclerotiorum* with a control efficacy of 71–80% with two foliar applications of *B. cereus* (SC1-1). Treatment of soybeans with *B. subtilis* strains SB01 or SB24 in field trials produced significant decreases in disease severity (Zhang et al., 2011). Vinodkumar et al. (2017) showed that several strains of *B. amyloliquefaciens* and *B. cereus* could decrease symptoms of root rot in carnation by up to 88% when applied as a root dip prior to transplanting. In pot studies, several *Bacillus* spp. significantly reduced the disease severity of *S. sclerotiorum* in common beans and mustard plants (Sabate et al., 2018). However, more field scale studies to prove the efficacy of *Bacillus*-based products are needed to support recommendations of commercial use. There are several *Bacillus amyloliquefaciens*/*subtilis* strains marketed as biofungicide treatments for a range of fungal diseases, such as *Sclerotinia* [e.g., Serenade Optimum, (Bayer CropScience, St. Louis, MO, United States), Cease, (Bioworks Inc., Victor, NY, United States), and Amplitude, (Marrone Bio Innovations Inc., Davis, CA, United States)]. These products are either formulations of *Bacillus* cells or *Bacillus* cells and their spent fermentation media, or a complex mixture of plant-supportive biochemicals that the bacteria produce. Together, these products are registered for applications of *Sclerotinia* control across leafy and root vegetables, legumes, and oilseed crops.

*Actinobacteria*, including *Streptomyces* spp., are known to produce a range of antimicrobial secondary metabolites, such as many antibiotics used in human and veterinary medicine, and play important roles in soil and rhizosphere ecology (Demain, 2000). Many *Streptomyces* spp. form associations with plants, either by living as endophytes in the tissues of the plant or closely associating within the rhizosphere. This makes them promising targets for the development of agricultural products for disease control and plant growth promotion (Palaniyandi et al., 2013; Rey and Dumas, 2017). The commercial products Actinovate (Novozymes BioAg Ltd. Saskatoon, Canada) and Mycostop (Verdera Oy, Espoo, Finland) are formulations of *Streptomyces lydicus* WYEC 108 and *Streptomyces griseoviridis* K61, respectively. Applications for the Actinovate control of *S. sclerotiorum* or *S. minor* include soil applications for disease control on *Brassica* head and stem vegetables, leafy vegetables, and legume vegetables. Mycostop is not registered for *Sclerotinia* control, but studies suggest that it may offer some protection. In a field trial testing for the efficacy of several different biocontrol products in controlling *S. sclerotiorum*in soybean, Zeng et al. (2012a) showed that *S. lydicus* (applied as a soil drench of Actinovate) reduced the disease severity index by 30.8% and t the number of sclerotia in harvested soybeans by 93.8% relative to diseased control plots. Chen et al. (2016b) compared the efficacy of two *Streptomyces* strains, *S. exfoliatus*FT05W and *S. cyaneus* ZEA17I against *Streptomyces lydicus*WYEC108, isolated from Actinovate, for the control of *S. sclerotiorum* in lettuce. Under growth chamber conditions, they reported that all three strains provided some level of protection against disease, with *S. lydicus* outperforming the others. However, under field conditions, *S. exfoliates* FT05W and *S. cyaneus* ZEA17I reduced the disease incidence, whereas *S. lydicus*WYEC108 did not. This highlights the need to conduct disease assays under field conditions but also highlights that several other *Streptomyces* strains may have a potential for commercialisation as biocontrol products for the control of *Sclerotinia* disease.

#### Mycoviruses

Mycoviruses, or viruses of fungi, also show potential as new biocontrol agents for crop fungal pathogens, such as *Sclerotinia* (Xie and Jiang, 2014; Zhang et al., 2020). *Sclerotinia* can host various mycoviruses, such as ssRNA, dsRNA, and single-stranded circular DNA viruses (reviewed in Xie and Jiang, 2014). The DNA mycovirus *S. sclerotiorum* hypovirulence-associated DNA virus 1 (SsHADV-1) can infect and confer hypovirulence on *S. sclerotorium* (Yu et al., 2013). When leaves of *Arabidopsis thaliana* or *Brassica napus* were sprayed with SsHADV-1 viral particles followed by inoculation with *S. sclerotiorum*, the leaves displayed reduced lesion development. Further research on this mycovirus found that it could convert pathogenic *S. sclerotiorum* into a beneficial plant endophyte by downregulating the expression of key *S. sclerotinia* virulence genes such as those encoding cell-wall degrading enzymes or effector-like genes, for example, Ss-Cmu1, SsITL, and SsSSVP1 (Zhang et al., 2020). Interestingly, the SsHADV-1 infection of *S. sclerotiorum* not only affected the expression of *Sclerotinia* genes, but when the viral-infected *Sclerotinia* colonised plant tissues it also caused increased the expression of *B. napus* defence- and hormone-associated genes, coupled with enhanced plant growth (Zhang et al., 2020). The viral infection system was also tested in the field. Spraying hyphal fragments of a SsHADV-1 infected *S. sclerotiorum* strain in *B. napus* plants during early flowering reduced *Sclerotinia* stem rot disease severity by 30–67% (Zhang et al., 2020).

#### Biocontrol Consortia and Microbiome Management

Biocontrols are typically sold and applied as single microbial inoculants. There is, however, an increasing interest in the delivery of consortiums of beneficial microbes to promote plant health (Czajkowski et al., 2020). Studies on *Sclerotinia* are scarce, but there is evidence of the potential for this approach in disease control. For example, in pea, the co-delivery of *T. harzianum*, *B. subtilis*, and *Pseudomonas aeruginosa* resulted in reduced mortality when challenged with *S. sclerotiorum* (Jain et al., 2015). In another example, Bahkali et al. (2014) found the *C. minitans*, *T. viride*, and *T. hamatum*, applied as soil drenches, were more effective than *S. griseoviridis*, *B. subtilis*, or *Pseudomonas fluorescens* alone in *S. sclerotiorum* disease suppression in beans. However, the best observed results (100% disease suppression) were achieved with the combination of either *C. minitans* + *S griseoviridis* or *T. hamatum* + *S. griseoviridis*. This highlights the possibility that combinations of biocontrol agents that have differing modes of action may be more effective than any single biocontrol applied alone. While promising, this is an area that requires more study, as there are currently few studies that show the impact of combinations of biocontrol organisms.

The concept of delivering microbial consortia for plant health is moving beyond applying a handful of microbes and toward the delivery of optimised synthetic communities (French et al., 2021; Trivedi et al., 2021). This research area is still in its early stages, but several studies show promise for disease control. The challenge for application to control *Sclerotinia* diseases will be the establishment of consortia, potentially over a range of plant tissues, and their persistence over sometimes long crop growing periods, as is the case for many row crops. An alternate approach is to manipulate the crop microbiome toward one that supports plant health. There is evidence this can be achieved by modifying farm management practices (e.g., reducing agrichemical use, increasing crop diversity) (French et al., 2021). Again, this area of research is also in its infancy, and significant research gaps are needed to be addressed before this approach becomes a reality.

#### Biochemicals and Biologically Derived Pesticide Products

There is a significant and growing body of research exploring the ability of biologically derived pesticide products to inhibit the growth of plant pathogens, such as *Sclerotinia.* This includes, for example, the fermentation and production or extraction of antimicrobial compounds or lytic enzymes, plant growth promoters, or induced systemic resistance (ISR) inducing compounds (Figure 3; Trejo-Estrada et al., 1998; El-Tarabily et al., 2000; Conn et al., 2008; Prapagdee et al., 2008; Baharlouei et al., 2010; Alam et al., 2012; Froes et al., 2012; Mathys et al., 2012; Wu et al., 2015; Chen et al., 2016a; Vergnes et al., 2020). At this time, the vast majority of these studies are in the *in-vitro* stage, with few examples of pot-scale, *in-planta* studies, and even fewer progressing to field studies. However, most of these reports describe promising candidates that have shown the ability to inhibit the germination and growth of *Sclerotinia* species.

Another promising area of research is ribonucleic acid interference approaches for the exogenous application of ribonucleic acid molecules for fungal pathogen control (Das and Sherif, 2020; Kuo and Falk, 2020). The foliar application of double-stranded RNAs to the leaf surface of canola or Arabidopsis significantly decreased *S. sclerotiorum* disease development (McLoughlin et al., 2018). There were, however, differences in the efficacy of some molecules over the two plant species. The authors suggest that the discrepancies may be due to differences in leaf morphology or cell structure characteristics.

## Future Perspectives

The severity of disease and crop losses across a broad range of horticultural and broad-acre farming sectors suggests that new and effective control measures need to be developed against *Sclerotinia* pathogens. This could include registration and/or development of fungicide chemistries with an increased window of preventative activity to minimise yield loss, and new modes of action to delay/prevent fungicide resistance. Increasing the understanding of how biocontrols work will facilitate optimisation of their formulation and delivery and provide insight into screening for more efficient/potent strains of commercial potential. Further, opportunities exist to combine chemical fungicides and biocontrols, or multiple biocontrols, to afford multiple modes of action and/or synergistic or additive effects. Together, these approaches can be incorporated with farming cultural practices in a coordinated attack on *Sclerotinia*. An understanding of how these approaches function together will be critical for durable disease control. Lastly, opportunities exist for the genetic modification of certain crops to attain complete disease resistance against *Sclerotinia.*

## Author Contributions

CO’S and LT conceived the review topic and outline. CO’S, KB, and LT wrote the review. All the authors listed have made a substantial, direct, and intellectual contribution to the study and approved it for publication.

## Conflict of Interest

The authors declare that the research was conducted in the absence of any commercial or financial relationships that could be construed as a potential conflict of interest.

## Publisher’s Note

All claims expressed in this article are solely those of the authors and do not necessarily represent those of their affiliated organizations, or those of the publisher, the editors and the reviewers. Any product that may be evaluated in this article, or claim that may be made by its manufacturer, is not guaranteed or endorsed by the publisher.
